# Azithromycin: An Underappreciated Quinolone-Sparing Oral Treatment for *Pseudomonas aeruginosa* Infections

**DOI:** 10.3390/antibiotics11040515

**Published:** 2022-04-13

**Authors:** Erlinda R. Ulloa, George Sakoulas

**Affiliations:** 1 Department of Pediatrics, School of Medicine, University of California Irvine, Irvine, CA 92697, USA; 2 Division of Infectious Disease, Children’s Hospital of Orange County, Orange, CA 92868, USA; 3 Sharp Rees-Stealy Medical Group, San Diego, CA 92123, USA; gsakoulas@health.ucsd.edu; 4 Collaborative to Halt Antibiotic-Resistant Microbes (CHARM), Department of Pediatrics, School of Medicine, University of California San Diego, La Jolla, CA 92093, USA

**Keywords:** multidrug-resistant Gram-negative infections, azithromycin, *Pseudomonas aeruginosa*, sinusitis, skin infection, otitis media

## Abstract

Outpatient treatment of *Pseudomonas aeruginosa* infections is challenged by increasing rates of resistance to fluoroquinolones, the only class of antibiotics which offers an established oral route of administration against this organism. Azithromycin does not demonstrate activity against *P. aeruginosa* when evaluated under standard methods of susceptibility testing with bacteriologic media. However, growing evidence shows that azithromycin is very active against *P. aeruginosa* when using physiologic media that recapitulate the in vivo milieu and is supported by animal models of infection and various clinical settings, including cystic fibrosis. We present three cases of outpatient management of *P. aeruginosa* otolaryngological infections successfully treated with oral azithromycin, 500 mg daily ranging from 3–8 weeks, where use of fluoroquinolones was not possible due to either resistance or patient intolerance. We review the previous data supporting this clinical approach, in the hope that this will alert clinicians to this treatment option and to inspire a more thorough clinical trial evaluation of azithromycin in this environment of growing medical need.

## 1. Introduction

The rapid spread of antibiotic resistance and high treatment failures in Gram-negative infections has spurred interest in the repurposing of drugs as therapeutic alternatives. One promising agent is the widely prescribed antibiotic azithromycin. Historically, this drug has not been considered in the management of multidrug-resistant Gram-negative infections simply because it has no activity against these pathogens when tested in Mueller–Hinton broth—the gold standard media used for antimicrobial susceptibility testing in clinical laboratories across the globe. Nonetheless, we have provided the first reports of azithromycin bactericidal activity in mammalian tissue culture media against multidrug-resistant strains of *Pseudomonas aeruginosa*, *Klebsiella pneumoniae*, *Acinetobacter baumannii*, *Stenotrophomonas maltophilia*, and *Achromobacter xylosoxidans*—pathogens for which suboptimal therapy may have catastrophic clinical consequences [1,2,3].

These therapeutic benefits partial to mammalian tissue culture media may be somewhat explained by the fact that Mueller–Hinton broth—which is composed of beef extract, casein and starch—is far from representative of the human physiological environment where antibiotics actually exert their activities. Standard antimicrobial susceptibility testing paradigms also overlook a key component in the clearance of any infection—the host immune system. Azithromycin has known immunomodulatory and anti-inflammatory properties [4,5,6]. Building on this literature, our group has uncovered surprising synergistic interactions between azithromycin and innate immune components. Specifically, azithromycin has been shown to sensitize multidrug-resistant Gram-negative pathogens to host immune clearance by antimicrobial peptides, neutrophils, and human serum. These azithromycin-related bactericidal effects have also been recapitulated in murine models of lung infection [1,2]. Yet, perhaps our most compelling data of azithromycin bactericidal activity comes from the clinical translation of our findings. Here we report our clinical experience using azithromycin in three patients with *P. aeruginosa* infections.

## 2. Clinical Cases

### 2.1. Case 1

A male in his 60s developed bilateral ear pressure and drainage. Clinical history was pertinent for chronic ear pruritis and cerumen for which he used a metal pick to gently scratch his ear canals. There was no prior antibiotic history. The patient was evaluated by otolaryngology and underwent bilateral ear lavage. He was prescribed otic ofloxacin but developed a contact allergy and the drops were discontinued. Ear canal cultures were positive for methicillin-resistant *Staphylococcus aureus* (MRSA) and extensively drug-resistant (XDR) *P. aeruginosa* (Table 1). He denied having any fevers, chills, or headaches. He had had no international travel. He initially felt better, but symptoms recurred. Systemic antibiotics were prescribed (but never started), and he was referred to infectious diseases. Examination revealed an edematous right ear canal resulting in almost 50% occlusion with purulent drainage. The left ear canal had some purulent drainage but was not swollen. The patient was started on minocycline 100 mg PO every 12 h and azithromycin 500 mg PO every 24 h. Otolaryngology also compounded and prescribed a 2 to 3-week course of polymyxin ear drops. The ear purulence diminished somewhat but the right ear canal remained swollen. Oral antibiotics were continued. A CT scan showed no evidence of mastoiditis. Over the subsequent weeks the purulence resolved and the edema slowly improved. Total oral antibiotic therapy was continued for 8 weeks. He was strongly advised not to insert Q-tips or other objects in his ear canals. The patient showed no signs of relapse at 90 days after completion of therapy.

### 2.2. Case 2

A female in her 60s with diabetes mellitus, hypertension, and hypercholesterolemia was referred to infectious diseases for a 3-year history of recurrent sinusitis refractory to antibiotics. Symptoms would resolve with antibiotics but then recur. Within the past year, sinus cultures were positive for *S. maltophilia*, *Staphylococcus epidermidis*, and most recently, pan-susceptible *P. aeruginosa*. Symptoms included sinus pressure, congestion, and vertigo. No fevers, chills, or systemic symptoms other than fatigue due to lack of sleep from the above symptoms occurred. While the *P. aeruginosa* was susceptible to all reported antibiotics (Table 1), to minimize the risk of *Clostridioides difficile*, azithromycin 500 mg PO daily was prescribed. After 2 weeks, the vertigo resolved, and symptoms improved. Azithromycin was continued for 21 days with durable relief. Of note, due to the recurrent nature of the sinusitis, an immune workup was performed and revealed a lambda light chain in serum protein electrophoresis and the patient was referred to hematology.

### 2.3. Case 3

A female in her 60s was referred with a history of extranodal sinus lymphoma and recurrent sinusitis over several years secondary to methicillin-susceptible *S. aureus* (MSSA), MRSA, and *P. aeruginosa*. The most recent episode of sinusitis was due to *P. aeruginosa*, treated with levofloxacin. Symptoms of sinus pressure and pain returned, now yielding a quinolone-resistant *P. aeruginosa* (Table 1). The patient was subsequently treated with a 21-day course of azithromycin 500 mg PO daily with symptomatic improvement.

## 3. Discussion

Infections due to *P. aeruginosa* are becoming increasingly difficult to treat due to increases in antimicrobial resistance. Bacterial sinusitis and otitis are generally managed in the outpatient setting with oral antibiotics. However, when these infections are caused by *P. aeruginosa*, the only established oral antibiotics are fluoroquinolones, which are becoming increasingly more difficult to deploy, not only because of rising rates of resistance but also the growing list of FDA warnings against their use. These include: (i) high propensity to cause *Clostridioides difficile* infection; (ii) concerns surrounding glycemic control in patients with diabetes mellitus; (iii) risks of tendon rupture, which is augmented further with concomitant glucocorticoids; (iv) risks of aortic rupture in patients with underlying cardiovascular disease. Furthermore, fluoroquinolone use is not favorable in the pediatric populations and in pregnancy except in cases of last resort [7,8,9,10].

Based on previous science published by our group and others, we successfully deployed azithromycin therapy to three patients with *P. aeruginosa* otolaryngological infections (two with sinusitis and one with otitis externa) where fluoroquinolones could not be used due to either resistance or patient allergy or intolerance. In the case of otitis externa, the XDR *P. aeruginosa* strain was not amenable to even the newest available antibiotics. In all cases the treatment was azithromycin 500 mg daily, with duration ranging from 3 weeks for the two monomicrobial sinusitis infections to 8 weeks for the polymicrobial otitis externa, which required concomitant minocycline to cover the MRSA. The 8-week duration for the otitis externa was based on bimonthly examinations until clinical resolution.

The patients were all in their 60s, with varying degrees of innate immune compromise due to comorbidities. It is notable that age 60 likely represents a pivotal period of innate immune system senescence in the human population, evidenced by the abrupt rise in incidence and mortality of respiratory and invasive infections in patients >60 years of age [11]. Indeed, the mortality seen over the past 2 years during the COVID-19 pandemic was no exception to this pattern [12].

Azithromycin is a well-established and safe antibiotic that has been available for decades. Its role in the chronic management of patients with cystic fibrosis has been largely attributed to its anti-inflammatory effect [6]. As prior work has shown and as these cases illustrate, a component of the anti-inflammatory effect may be due to its direct anti-pseudomonal activity. In comparison to fluoroquinolones, azithromycin poses a lower risk of *C. difficile*, has a more established track record in pregnancy and pediatrics, and does not carry a risk of tendon and aortic rupture. It is notable that there is a risk for QTc prolongation, particularly in patients on anti-arrhythmic medications, but that was not a concern in our patients [13].

The therapeutic success seen in our patients is likely multifactorial and related to in vivo physiological and immunomodulatory properties that are difficult to capture in our current in vitro antimicrobial susceptibility testing paradigms. Indeed, strain specific genome sequences have been used to predict and demonstrate that bacterial metabolic responses vary based on differences in media or nutritional environments that, in turn, may affect the virulence and expression of antibiotic resistant genes [14,15]. Differences in cation concentrations and bicarbonate levels have been shown to influence antibiotic susceptibility results [16,17]. Bicarbonate levels can affect the proton motive force of bacteria that can, in turn, affect the activity and/or uptake of antibiotics such as azithromycin that work intracellularly to inhibit protein synthesis [17]. Azithromycin also has robust immunomodulatory and anti-inflammatory properties that have proven beneficial clinically [5,6]. In patients with cystic fibrosis, for example, azithromycin has been shown to improve lung function and reduce *P. aeruginosa* exacerbations [5,6]. Azithromycin has also been shown to work synergistically with bacterial pore-forming components of the innate immune system, such as serum complement and antimicrobial peptides, that facilitate the entry of drugs like azithromycin that work intracellularly [1,2,3]. The clinical efficacy of azithromycin is also likely complemented by its ability to impair bacterial biofilm synthesis [3,18,19], and the attenuation of other bacterial virulence factors [19,20,21], including adherence to host epithelial cells [22,23].

While not described in this case series in order to keep infection types more homogeneous, we wish to inform readers that we have successfully utilized azithromycin in multidrug-resistant *P. aeruginosa* soft tissue infections, including a patient in his 40s with *P. aeruginosa* Fournier’s gangrene in the setting of neutropenia from myelodysplastic syndrome. Alongside aggressive source control, the patient had a satisfactory outcome with piperacillin-tazobactam 4.5 g IV every 6 h plus azithromycin 500 mg IV daily, despite a minimum inhibitory concentration to piperacillin-tazobactam of 64 mg/L and resistance to other available agents (this was before the availability of ceftazidime-avibactam and subsequent agents).

*P. aeruginosa* susceptibility to azithromycin was not confirmed in the reported cases. In our experience, azithromycin is universally “resistant” when tested via standard microdilution methods in Mueller–Hinton broth, and not reflective of in vivo activity. Nonetheless, there is mounting evidence to suggest that *P. aeruginosa* is susceptible to azithromycin when its activity is tested under more physiological conditions (e.g., mammalian tissue culture media), even across XDR strains [1,24,25]. Moreover, serial passage of *P. aeruginosa* (over 10 consecutive days) at sub-minimum inhibitory concentrations of azithromycin in physiologic media demonstrate no increased resistance to azithromycin over this time frame [1]. We suspect that the majority of *P. aeruginosa* are indeed susceptible to azithromycin killing. However, larger scale studies are needed to determine if these findings are universal and to evaluate the possibility of resistance development over time. We hope that continued studies will lead to further development of what we believe to be more accurate assessments of antibiotic activity in vitro under more physiological conditions that recapitulate the in vivo environment, as compared to current bacteriological media that is based on supporting viable bacterial growth.

## Figures and Tables

**Table 1:** Broth microdilution minimum inhibitory concentrations (MICs) for *P. aeruginosa* from strains isolated from ear canal (case 1) and sinuses (case 2 and case 3).

Antibiotic	Case 1		Case 2		Case 3	
	MIC (mg/L)	Interpretation	MIC (mg/L)	Interpretation	MIC (mg/L)	Interpretation
Amikacin	>32	R	-	-	-	-
Aztreonam	>16	R	≤4	S	≤4	S
Cefepime	>16	R	≤2	S	8	S
Cefidericol	8	I	-	-	-	-
Ceftazidime	>16	R	≤1	S	4	S
Ceftazidime/avibactam	>16	R	-	-	-	-
Ceftolozane/tazobactam	>8	R	-	-	-	-
Ciprofloxacin	>2	R	≤0.25	S	>2	R
Delafloxacin	>2	R	-	-	-	-
Colistin	2	I	-	-	-	-
Eravacycline	4	(ND)	-	-	-	-
Gentamicin	>8	R	≤2	S	>8	R
Imipenem	>16/4	R	-	-	>8	R
Levofloxacin	>4	R	≤0.50	S	>4	R
Meropenem	>8	R	≤1	S	2	S
Meropenem/vaborbactam	>16/8	R	-	-	-	-
Piperacillin/tazobactam	>64	R	≤8	S	≤8	S
Tobramycin	>8	R	≤2	S	≤2	S

Minimum inhibitory concentrations (MICs) were determined as per Clinical Laboratory Standards Institute (CLSI) guidelines in Mueller–Hinton broth. I, intermediate; ND, not determined; R, resistant; S, susceptible.

## Data Availability

Not applicable.
